# Adherence of denosumab treatment for low bone mineral density in Japanese people living with HIV: a retrospective observational study

**DOI:** 10.1186/s40780-023-00315-9

**Published:** 2023-12-07

**Authors:** Yusuke Kunimoto, Ryosuke Matamura, Hiroshi Ikeda, Hiroto Horiguchi, Satoshi Fujii, Masayoshi Kobune, Masahide Fukudo, Takaki Toda

**Affiliations:** 1 Department of Pharmacy, Sapporo Medical University Hospital, Sapporo, 060-8543 Japan; 2 Department of Hematology, Sapporo Medical University School of Medicine, Sapporo, Japan; 3 Department of Clinical Pharmacology, Faculty of Pharmaceutical Sciences, Hokkaido University of Science, Sapporo, Japan

**Keywords:** Denosumab, Adherence, HIV, Osteoporosis, COVID-19

## Abstract

**Background:**

Long-term care issues, specifically metabolic bone disorders, are a concern for people living with human immunodeficiency virus (PLWH) who undergo life-long antiretroviral therapy (ART). Previous clinical trials with denosumab, an anti-RANKL antibody inhibitor, have revealed its effectiveness in increasing bone mineral density (BMD) in patients with osteoporosis. However, there are limited data on adherence and effectiveness of denosumab treatment for osteoporosis in PLWH. Hence, this study aimed to investigate the adherence and effectiveness of denosumab treatment for osteoporosis in Japanese PLWH.

**Methods:**

This study is a retrospective exploratory analysis of 29 Japanese PLWH who initiated denosumab treatment for osteoporosis, between 2013 and 2021. The study included patients who received at least one dose of denosumab every 6 months. Adherence and persistence were defined as receiving two consecutive injections of denosumab 6 months ± 4 weeks apart and 6 months + 8 weeks apart, respectively. The primary outcome measure of the study was the adherence of denosumab treatment for 24 months. The secondary outcome measures included treatment persistence and BMD. The period after January 2020 was defined as the coronavirus disease 2019 (COVID-19) pandemic period, and its impact on adherence was investigated.

**Results:**

The treatment adherence rates at 12 and 24 months were 89.7% and 60.7%, respectively. By contrast, the treatment persistence at 12 and 24 months was 100% and 85.7%, respectively. More patients in the group who initiated denosumab treatment after the COVID-19 pandemic reached non-adherence than in the group who initiated denosumab treatment before the pandemic. BMD at the lumbar spine and femoral neck significantly increased compared to that at baseline, with median percentage changes of 8.7% (*p* < 0.001) and 3.5% (*p* = 0.001), respectively.

**Conclusions:**

The results showed that patients in the study had a high rate of non-adherence but a lower rate of non-persistence. Additionally, PLWH on ongoing ART experienced increased BMD with denosumab treatment. This study provides an opportunity to improve future strategies for denosumab treatment in the Japanese PLWH.

**Supplementary Information:**

The online version contains supplementary material available at 10.1186/s40780-023-00315-9.

## Background

The prognosis of human immunodeficiency virus (HIV) infection has improved drastically. However, lifelong antiretroviral therapy (ART) is required to maintain its therapeutic effects, and issues related to long-term care have emerged [1]. In particular, metabolic bone disorders in people living with HIV (PLWH) have received notable attention in recent years [2]. There is a higher proportion of PLWH with decreased bone mineral density (BMD) than in non-infected individuals [3]. In addition, a meta-analysis of BMD reduction found that the risk of BMD reduction was higher in the ART group than in the untreated group [4]. Other studies have reported that the antiretroviral drug (ARV), tenofovir (TFV) can cause BMD reduction [5, 6]. Therefore, the pharmacotherapy of osteoporosis would be an important strategy for preventing fractures in PLWH. Specifically, the effectiveness of bisphosphonates and denosumab, an anti-RANKL antibody, has been reported in non-HIV-infected elderly women [7, 8].

Bisphosphonates, the prevalent treatment for osteoporosis, have been reported to improve BMD in PLWH [9, 10]. However, bisphosphonates have also been associated with difficulties in maintaining medication adherence [11]. Treatment with denosumab has been shown to significantly increase BMD in clinical trials and a reduction in fracture risk has been reported, particularly for the treatment of postmenopausal osteoporosis in women [8]. Compared with bisphosphonates, denosumab has high treatment adherence and satisfaction rates [12, 13]. However, there have been multiple reports of a decrease in adherence to denosumab being related to a decrease in the treatment efficacy [14–16], and adherence to denosumab is thought to be an important indicator in osteoporosis treatment. Self-administration of denosumab is not allowed in Japan. Therefore, regular appointment scheduling and hospital visits are crucial to maintain adherence to the subcutaneous injections of denosumab. To date, no reports have clarified the adherence to denosumab in PLWH, and there are only a few reports on the effectiveness of the treatment [17, 18].

Adherence to denosumab treatment is believed to be affected by accessibility to healthcare [19]; thus, it is important to examine local adherence data in countries where PLWH are present. Additionally, the confusion related to healthcare access caused by the coronavirus disease 2019 (COVID-19) pandemic from January 2020 onward has led to a decrease in patient visits [20, 21]; we hypothesized that the COVID-19 pandemic may have affected denosumab treatment adherence in PLWH. The objective of this study was to examine the adherence to denosumab treatment in Japanese PLWH who have osteoporosis. Moreover, we assessed the impact of the COVID-19 pandemic on denosumab treatment adherence in PLWH and the changes in BMD associated with denosumab treatment.

## Methods

### Study design and patients

This retrospective exploratory study was conducted on Japanese PLWH aged 20 years or older who initiated denosumab treatment for osteoporosis between June 1, 2013, and June 30, 2021, at Sapporo Medical University Hospital. We screened 123 PLWH who had visited our hospital and selected those who had initiated denosumab treatment for osteoporosis. All eligible patients who received at least one dose of denosumab (60 mg administered as a single subcutaneous injection every 6 months) were enrolled in the study. The data to be used for analysis were collected until August 31, 2022. The Subject Enrollment Flowchart is presented in Supplemental Figure S1. This study was approved by the institutional review board of Sapporo Medical University (342–141). Patient consent was obtained using the opt-out method.

### Data collection

All data were collected from the patients’ hospital charts. Demographic and clinical data included age, sex, body mass index, smoking status, date of denosumab administration, date of BMD measurement, history of acquired immunodeficiency syndrome diagnosis, duration of HIV diagnosis, duration of ART, ART regimen, concomitant medications, and laboratory data including serum calcium levels, serum albumin levels, HIV-RNA viral levels, and CD4 cell counts. The BMD of the lumbar spine and femoral neck were measured using dual-energy X-ray absorptiometry (Horizon A DXA System; HOLOGIC, Waltham, MA, USA).

### Outcome measures

The primary outcome was adherence to denosumab treatment for 24 months. Secondary outcomes included adherence to denosumab treatment at 12 months after treatment, the first incidence of non-adherence and non-persistence with denosumab treatment initiation, comparison of denosumab adherence before and after the COVID-19 pandemic, persistence with denosumab treatment at 12 and 24 months after treatment initiation, and changes in the lumbar spine and femoral neck BMD from baseline to the follow-up evaluation. Safety outcomes were assessed for hypocalcemia and infectious events. Hypocalcemia was defined as a total serum calcium level < 8.4 mg/dL. Infectious events were defined using physician diagnosis documented in the electronic medical record.

### Definitions

Adherence was defined as receiving two consecutive injections of denosumab 6 months ± 4 weeks apart [12, 19, 22]. Persistence was defined as receiving two consecutive in denosumab injections 6 months + 8 weeks apart [19, 22]. As previously reported, the medication coverage ratio (MCR) was calculated as the proportion of time a patient's treatment was covered by denosumab [22]. A single injection of denosumab was assumed to provide 6 months of medication coverage. The period after January 2020 was defined as after the COVID-19 pandemic period. Virological suppression was defined as an HIV-1 RNA level of < 50 copies/mL. A serum calcium concentration < 8.5 mg/dL was defined as hypocalcemia. We corrected the total serum calcium levels by serum albumin levels if albumin levels < 4.0 g/dL, using the following formula: corrected calcium (mg/dL) = total calcium (mg/dL) + (4.0 − albumin [g/dL]) [23]. Infectious adverse events (AEs) were defined as symptoms of infection for which systemic antimicrobials were prescribed. To analyze changes in BMD, we calculated the annual percentage change from baseline to follow-up using the following formula: {[(BMD follow-up—BMD baseline) / BMD baseline] * 100 / days between assessments} × 365.25 [24]. Patients were divided into two groups based on a 3% annual increase in BMD [25, 26].

### Statistical analyses

We did not perform formal sample size calculations because our primary endpoint was the assessment of adherence to denosumab therapy within a single group without specific targets or comparisons, given the exploratory nature of this study. Continuous variables were presented as medians and interquartile ranges (first and third quartiles). Categorical variables were expressed as the number of patients and percentages. Statistical analysis was performed using the Mann–Whitney U test for continuous variables and Fisher’s exact test for categorical variables. BMD comparisons during the study period were performed using the Wilcoxon signed-rank test. Kaplan–Meier survival analysis was used to assess adherence and persistence, and log-rank tests were used to compare patients who initiated denosumab treatment before and after the COVID-19 pandemic. Annualized lumbar spine and femoral neck BMD changes and medication coverage ratio at follow-up were analyzed using Spearman's correlation analysis. Because the proportion of missing data was minimal, we conducted each analysis by excluding cases with missing data. Statistical significance was defined as *p* < 0.05 for a two-sided test. All analyses were performed using JMP Pro version 15 (SAS Institute, Cary, NC, USA).

## Results

### Characteristics of patients

Twenty-nine PLWH who received denosumab were included in the analysis. One patient with missing data on follow-up BMD was excluded from the analysis of changes in the lumbar spine and femoral neck BMD. Another patient was lost to follow-up because of relocation at 966 days after initiating denosumab treatment. The median follow-up duration of all patients was 1417 (range, 506–3755) days. The baseline demographic and clinical characteristics of patients enrolled in this study are shown in Table 1. All patients were virologically suppressed, with a median ART duration of 4 years; 23 patients (79.3%) used TFV.

**Table 1:** Baseline demographics and clinical characteristics of patients^a^

Characteristics	
Number of patients	29
Age (years), median (IQR)	39.0 (35.5–44.0)
Gender, male, n (%)	26 (89.7)
BMI (kg/m^2^), median (IQR)	20.2 (18.7–23.6)
Current smoking, n (%)	12 (41.4)
Prior AIDS diagnosis, n (%)	10 (34.5)
Time since diagnosis HIV (years), median (IQR)	4 (1–9)
HIV-RNA < 50 copies/mL, n (%)	29 (100)
CD4 cell count (cells/µL), median (IQR)	438 (301–805)
Time on antiretroviral therapy (years), median (IQR)	4 (1–8)
Backbone drug	
TAF/FTC, n (%)	13 (44.8)
TDF/FTC, n (%)	10 (34.5)
ABC/3TC, n (%)	6 (20.7)
Key drug class	
INSTI, n (%)	18 (62.1)
PI, n (%)	10 (34.5)
NNRTI, n (%)	1 (3.4)
Number of non-HIV medications, median (IQR)	2 (1–4)
Use of ≥ 5 non-HIV medications, n (%)	1 (3.4)
Serum-corrected Ca (mg/dL), median (IQR)	9.2 (9.0–9.4)
BP pre-treatment, n (%)	6 (20.7)
Lumbar spine BMD (g/cm^2^), median (IQR)	0.798 (0.753–0.873)
Femoral neck BMD (g/cm^2^), median (IQR)	0.606 (0.570–0.660)

*Abbreviations*: *3TC* lamivudine, *ABC* abacavir, *AIDS* acquired immunodeficiency syndrome, *BMD* bone mineral density, *BP* bisphosphonate, *BMI* body mass index, *Ca* calcium, *FTC* emtricitabine, *INSTI* integrase strand transfer inhibitor, *NNRTI* non-nucleoside reverse transcriptase inhibitor, *NRTI* nucleoside reverse transcriptase inhibitor, *PI* protease inhibitor, *TAF* tenofovir alafenamide fumarate, and *TDF* tenofovir disoproxil fumarate

^a^ Data are expressed as numbers and frequencies (%) or median values with interquartile ranges (IQRs)

### Adherence and persistence

**Fig. 1:**
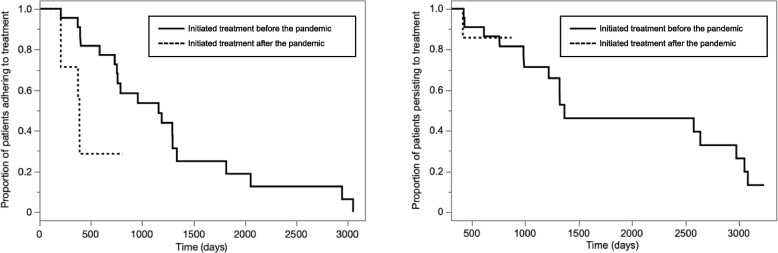
Kaplan–Meier survival curves for patients initiating denosumab treatment before and after the COVID-19 pandemic. Kaplan–Meier survival curves of denosumab treatment adherence and persistence in 29 patients comparing patients who initiated treatment before (solid line) and after (dotted line) the COVID-19 pandemic. Patients who initiated denosumab before the pandemic showed a significantly lower risk of non-adherence than those who initiated treatment after the pandemic (log-rank test, *p* = 0.012). Denosumab treatment persistence was not significantly different between patients who initiated treatment before and those who initiated treatment after the pandemic (log-rank test, *p* = 0.976). COVID-19, coronavirus disease 2019

Adherence rates at 12 and 24 months were 89.7% and 60.7%, respectively. The median time from the commencement of denosumab treatment to non-adherence was 957 days (95% CI, 404–1296 days) (Supplemental Figure S2). The persistence rates at 12 and 24 months were 100% and 85.7%, respectively. The median persistence was 1366 days (95% CI, 992–2973 days) (Supplemental Figure S2). A comparison of the demographic and clinical characteristics between the denosumab treatment adherent and non-adherent groups at 24 months is shown in Table 2. The durations of HIV diagnosis and ART in the adherence group were significantly longer than those in the non-adherence group (*p* = 0.007 and *p* = 0.005, respectively). The proportion of patients who initiated denosumab treatment before the pandemic was significantly higher in the adherent group (*p* = 0.022). The Kaplan–Meier survival curves of denosumab adherence and persistence divided into patients who initiated denosumab before and after the COVID-19 pandemic are shown in Fig. 1. Patients who initiated denosumab after the pandemic had a shorter time to non-adherence than patients who initiated denosumab before the pandemic (*p* = 0.012). Persistence was not significantly different between patients who initiated denosumab treatment before and after the pandemic (*p* = 0.976). A comparison of the demographic and clinical characteristics between the denosumab treatment initiated before and after the COVID-19 pandemic groups is presented in Supplemental Table S1. Before the COVID-19 pandemic, the group had higher CD4 cell counts (*p* = 0.012) and a significantly longer duration of ART (*p* = 0.022).

**Table 2:** Demographic and clinical characteristics of denosumab adherence versus non-adherence groups at 24 months (*n* = 28) ^a^

Characteristics	Denosumab treatment adherence at 24 months		*p*-value ^c^
	Adherent group (*n* = 17) ^b^	Non-adherent group (*n* = 11) ^b^	
Age (years), median (IQR)	39.0 (36.0–43.3)	39.0 (34.0–45.0)	0.962
Gender, male, n (%)	15 (88.2)	10 (90.9)	1.000
BMI (kg/m^2^), median (IQR)	21.4 (19.2–24.7)	19.0 (18.6–20.7)	0.145
Current smoking, n (%)	6 (35.3)	6 (54.5)	0.441
Prior AIDS diagnosis, n (%)	4 (23.5)	6 (54.5)	0.125
Time since diagnosis HIV (years), median (IQR)	5 (1–10)	1 (0–4)	0.007
CD4 cell count (cells/µL), median (IQR)	534 (373–805)	355 (149–827)	0.158
Time on antiretroviral therapy (years), median (IQR)	4 (2–10)	1 (1–3)	0.005
Denosumab treatment initiated before the COVID-19 pandemic, n (%)	16 (94.1)	6 (54.5)	0.022

*Abbreviations*: *AIDS* acquired immunodeficiency syndrome, *BMI* body mass index, *COVID-19* coronavirus disease 2019, *HIV* human immunodeficiency virus, *IQR* interquartile range

^a^ Data are expressed as numbers and frequencies (%) or median values with interquartile ranges (IQRs)

^b^ Adherence and persistence were evaluated for 24 months, excluding one case that did not reach 24 months after the commencement of denosumab treatment

^c^ Fisher’s exact test was used for categorical data, and the Mann–Whitney U test was used for continuous data

### BMD changes with denosumab treatment

**Fig. 2:**
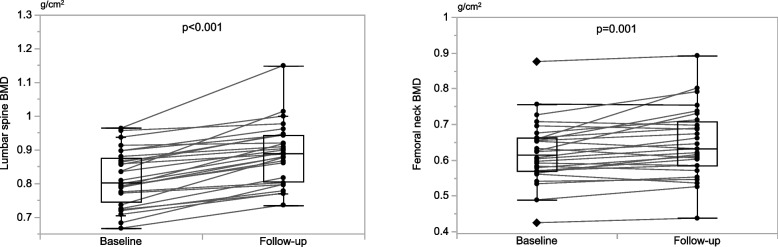
Box-and-whisker plots and Wilcoxon signed-rank tests of baseline versus follow-up bone mineral density. One patient who did not undergo follow-up BMD measurements was excluded from the analysis. Box-and-whisker plots show the median and interquartile ranges, as well as the upper and lower extremes. Diamond-shaped dots show outliers. Dot plots depict the baseline and follow-up BMD in 28 paired samples connected by a line. Paired Wilcoxon signed-rank tests were used to evaluate changes in BMD. A *p*-value < 0.05 indicated statistical significance. The follow-up lumbar spine BMD was significantly higher than that at baseline (median, 0.803 [IQR, 0.744–0.876] versus 0.888 [0.805–0.943]), respectively. Similarly, the follow-up femoral neck BMD was significantly higher than that at baseline (median, 0.615 [IQR, 0.569–0.661] versus 0.631 [0.584–0.708]), respectively. The median follow-up time was 658 (IQR, 501–906) days. BMD, bone mineral density; IQR, interquartile range

The BMD changes from baseline to follow-up with denosumab treatment are shown in Fig. 2. The follow-up lumbar spinal BMD significantly increased compared to baseline (median percentage changes, + 8.7%, *p* < 0.001). All patients showed increased lumbar spine BMD at follow-up. The follow-up femoral neck BMD significantly increased compared to baseline (median percentage changes, + 3.5%, *p* = 0.001). The median annualized lumbar spine and femoral neck BMD changes were + 4.2% (95% CI, 2.4–6.1) and + 1.7% (95% CI, -0.8–2.8), respectively. Annualized lumbar spine and femoral neck BMD changes were not significantly associated with adherence status at 24 months or MCR to follow-up (Supplemental Figures S3, S4). Seventeen patients (60.7%) in the lumbar spine and six patients (21.4%) in the femoral neck showed a significant increase of > 3%. Patients were divided into an annual increase group of > 3% and a non-increased group based on BMD at follow-up. To investigate the factors contributing to the BMD increase, a comparison of the demographic and clinical characteristics was conducted between the increased and non-increased groups (Table 3). Compared to the non-increased group in lumbar spine BMD, the increased group had a lower median age (*p* = 0.018) and a higher proportion of patients using TFV (*p* = 0.022).

**Table 3:** Comparison of demographic and clinical parameters between increased and non-increased BMD groups ^a^

Parameters	Lumbar spine BMD		*p*-value ^c^	Femoral neck BMD		*p*-value ^c^
	Increased group (≥ 3% per year) *n* = 17 ^b^	Non-increased group (< 3% per year) *n* = 11 ^b^		Increased group (≥ 3% per year) *n* = 6 ^b^	Non-increased group (< 3% per year) *n* = 22 ^b^	
Age (years), median (IQR)	37.0 (34.5–40.5)	43.0 (39.0–46.0)	0.018	40.5 (32.3–45.0)	39.0 (35.0–43.3)	0.978
Gender, male, n (%)	15 (88.2)	10 (90.9)	1.000	6 (100)	19 (86.4)	1.000
Body mass index (kg/m^2^), median (IQR)	20.2 (18.9–23.8)	19.8 (17.9–23.7)	0.452	23.2 (18.4–23.7)	20.0 (18.6–23.8)	0.675
Current smoking, n (%)	9 (52.9)	2 (18.2)	0.115	1 (16.7)	10 (45.5)	0.355
Prior AIDS diagnosis, n (%)	4 (23.5)	5 (45.5)	0.409	2 (33.3)	7 (31.8)	1.000
Time since diagnosis HIV (years), median (IQR)	1 (1–6.5)	4 (1–12)	0.325	1 (0.75–6.8)	4 (1–9)	0.458
CD4 cell count, median (IQR)	394 (301–545)	766 (149–907)	0.132	518 (180–782)	436 (306–804)	0.716
Time on antiretroviral therapy (years), median (IQR)	2 (1–5)	4 (1–9)	0.258	2 (1–5)	4 (1–8)	0.492
TFV use at baseline, n (%)	16 (94.1)	6 (54.5)	0.022	5 (83.3)	17 (77.3)	1.000
BP pre-treatment, n (%)	1 (5.9)	4 (36.4)	0.062	0 (0)	5 (22.7)	0.553
Lumbar spine BMD at baseline (g/cm^2^), median (IQR)	0.789 (0.723–0.845)	0.867 (0.774–0.912)	0.105	0.827 (0.769–0.883)	0.800 (0.732–0.874)	0.801
Femoral neck BMD at baseline (g/cm^2^), median (IQR)	0.606 (0.574–0.679)	0.623 (0.540–0.654)	0.621	0.602 (0.568–0.655)	0.615 (0.568–0.680)	0.654
Denosumab treatment initiated before the COVID-19 pandemic, n (%)	13 (76.5)	8 (72.7)	1.000	5 (83.3)	16 (72.7)	1.000
Adherence at 12 months, n (%)	14 (82.4)	11 (100)	0.258	6 (100)	19 (86.4)	1.000
Adherence at 24 months, n (%)	12 (70.6)	6 (54.5)	0.444	5 (83.3)	13 (59.1)	0.375
MCR to follow-up (%), median (IQR)	95.0 (92.3–98.9)	98.6 (93.2–99.1)	0.525	95.6 (91.6–99.3)	95.2 (92.4–99.1)	0.889

*Abbreviations*: *AIDS* acquired immunodeficiency syndrome, *BMD* bone mineral density, *BMI* body mass index, *BP* bisphosphonate, *COVID* coronavirus disease, *HIV* human immunodeficiency virus, *IQR* interquartile range, *MCR* medication coverage ratio, *TFV* tenofovir

^a^ Data are expressed as numbers and frequencies (%) or median values with IQR

^b^ Patients who did not undergo follow-up BMD measurements were excluded from the analysis

^c^ Fisher’s exact test was used for categorical data, and the Mann–Whitney U test was used for continuous data

### Safety outcomes

Six patients (20.7%) developed hypocalcemia. Of these, five patients were classified as grade 1 and one patient was classified as grade 2. The incidence of infectious AEs was 24.1% (seven patients), including skin infections in three patients, urinary tract infections in two patients, gastrointestinal infections in one patient, and upper respiratory infections in one patient. However, none of the patients had severe infections requiring intravenous antimicrobial treatment.

## Discussion

To our knowledge, this is the first study to clarify the adherence of PLWH to denosumab in a clinical setting. In this study, we obtained two main findings. First, the adherence rate to denosumab treatment among PLWH was 89.7% at 12 months but decreased substantially at 24 months. Second, the BMD of the PLWH receiving ART was improved with denosumab treatment.

In PLWH, adherence to denosumab was maintained at a high rate of 89.7% at 12 months but decreased to 60.7% by 24 months. There have been many reports of tests measuring adherence in postmenopausal osteoporosis patients, with rates of 38–100% and 22–99% at 12 and 24 months, respectively [19, 22, 27–30]. In a previous study, the adherence rates to denosumab at 12 and 24 months were 100% and 99% respectively; in comparison, our results showed lower denosumab treatment adherence than those in the aforementioned study [29]. The participants in the previous study were mostly older women, whereas those in our study were mostly younger men. In general, younger age and male sex have been identified as factors associated with lower adherence to treatment, which is consistent with the lower adherence results found in our study [28, 31]. However, persistence, defined as an 8-week tolerance range, has been reported to be between 56–84% and 40–72% at 12 and 24 months, respectively [19, 27, 28, 30–33]. In our study, persistence was maintained at high rates of 100% and 85.7% at 12 and 24 months, respectively, suggesting that persistence tended to be higher than that previously reported. In contrast to other reports, all patients in our study were receiving ART, which required them to regularly receive prescriptions for ARVs. According to a report in Japan, PLWH demonstrated higher adherence to ARVs [34], suggesting that Japanese PLWH may have a greater likelihood of maintaining regular outpatient visits. Therefore, fewer patients greatly delayed their appointments, which may have contributed to the maintenance of persistence. One study suggested that older adults who often have multiple chronic diseases and take many concomitant medications may have lower priorities for denosumab treatment for osteoporosis, leading to missed appointments [22]. In our study, because ARV prescriptions and denosumab treatment were administered at the same hospital, regular appointments were more likely to be maintained to receive ARVs even if the priority of osteoporosis therapy was low.

The results of our study showed that the non-adherence group at 24 months had a shorter medical history, shorter ART history, and a higher proportion of patients who initiated treatment after the COVID-19 pandemic. Patients with shorter medical and treatment histories may not be able to establish regular hospital visits and may delay denosumab administration. While there have been reports that younger PLWH do not adhere to clinical appointments, our results did not support the findings of a previous report [35]. This may have been attributed to the smaller number of patients and the narrower age range of participants in our study. Additionally, the relationship between the shortage of medical and ART history and non-adherence to denosumab may have been affected by the avoidance of hospital visits due to the COVID-19 pandemic. In our study, the 24-month adherence assessment period for patients who initiated denosumab treatment after 2018 overlapped with that after the pandemic, and it is possible that the avoidance of clinical visits had an effect on the decrease in treatment adherence. The Kaplan–Meier curve showed that patients who initiated denosumab treatment after the pandemic had non-adherence earlier than those who commenced treatment before the pandemic (Fig. 1). However, persistence did not appear to be affected by the COVID-19 pandemic. Because of limited numbers of events (i.e., non-adherence), only univariate analysis was possible in this study (Table 2). We consider that a multivariate analysis adjusting for variables (i.e., age, sex, and residential area [31]), potentially associated with adherence to denosumab therapy, would be necessary to further investigate the influence of the pandemic on the adherence. For example, patients living far from our hospital may have been more vulnerable to the pandemic's influence, leading to a greater decrease in the adherence. In our study, patients and healthcare providers seemed to have cooperated to minimize changes in clinical appointments. During infectious disease outbreaks, discussing methods to continue injection therapy without delay through regular visits is an important issue that can be applied to ART management with long-acting injectable agents.

This study suggested that denosumab treatment improved BMD in PLWH receiving ART. Previous studies have reported that the annual BMD increases with denosumab treatment ranging from BMDs of 3.4–5.8% for the lumbar spine and 1.4–3.7% for the femoral neck [17, 32, 33, 36–41]. The present study found similar BMD improvement rates. Additionally, this study found that a significant improvement in lumbar spine BMD was associated with lower age and use of TFV at baseline, but not with femoral neck BMD, whereas a previous study suggested that denosumab may be more effective in younger patients [42], which is supported by the results of this study. This study also found that the use of TFV at baseline was associated with improved lumbar spine BMD. A previous study on postmenopausal women with osteoporosis reported that patients with greater BMD loss at 2 years before initiating denosumab had greater BMD increases after commencing treatment [43]. This study also suggested that patients using TFV at baseline may have had a greater decline in BMD immediately before denosumab initiation, which may have led to a greater increase in BMD. The potential impact of concurrent use of TFV on BMD changes during denosumab treatment was not investigated. By contrast, our study did not find a significant association between denosumab adherence or MCR and BMD improvement. Our results might suggest that a short-term delay in denosumab administration may not affect the impact on the BMD increase; however, the MCR results in this study tended to be high overall and the range of observed values was narrow, which may have prevented an accurate understanding of the relationship with BMD change.

In this study, 39% of PLWH did not adhere to denosumab treatment when adherence was assessed at a permissible gap of 4 weeks based on 2-year evaluations. However, when durability was evaluated with an 8-week permissible gap, only 14% of the patients were non-adherent. These findings suggest that it is possible to continue denosumab treatment in Japan without significantly delaying the dosing interval. This study also found that denosumab treatment improved BMD in PLWH and that it may be a future treatment option for osteoporosis in this population. Additionally, no critical infections or low calcium levels were reported and there were no new safety concerns regarding the use of denosumab in PLWH.

This study had some limitations. First, the sample size was small, limiting the generalizability of the conclusions. It was also impossible to control for potential confounding factors or eliminate their influence on the results. To address these limitations, it is necessary to design a multicenter collaborative study with a larger sample size and obtain results through multivariate analysis that accounts for adjusted confounding factors. Additionally, the study was conducted retrospectively, which means that the intervals between baseline and follow-up BMD measurements were not consistent. To address this issue, the study calculated the annual rate of change in BMD to facilitate the evaluation of treatment effects; however, this method assumes that the increase in BMD with denosumab treatment occurs linearly over time, which may not be the case. Several studies have reported that BMD increase with denosumab treatment persists over a long period without plateauing [44, 45]. However, the initial rate of increase in BMD after denosumab was high [44]. Therefore, there is a risk of overestimating the annual rate of increase in BMD if follow-up BMD measurements are taken soon after the initiation of treatment than at a longer interval between measurements. The findings may have limited generalizability to other age groups or female populations owing to the predominance of young male individuals in the study sample. Additionally, this study only included Japanese patients; therefore, the results may not be generalizable to other countries or ethnicities.

This study highlights the issue of reduced adherence to denosumab treatment among Japanese PLWH, providing an opportunity to review and improve future strategies for denosumab treatment in this population. One study found that longer intervals between doses of denosumab resulted in smaller BMD responses [46]; therefore, it is important to minimize the gaps between the scheduled 6-monthly dosing dates. The factors that impede medication adherence are multifaceted. Therefore, a multifactorial approach is needed to address them [47]. As one key approach, healthcare providers must understand the risks of BMD reductions and fractures associated with poor adherence to denosumab treatment and educate their patients accordingly. Positive feedback from patients based on increased BMD improves adherence to denosumab treatment [48]. Additionally, a temporary switch to oral bisphosphonates may be considered an option to avoid the risks of rapid decline in BMDs in cases where rescheduling of denosumab treatments is necessary, as reported in a previous study [49].

## Conclusions

We aimed to investigate denosumab adherence among Japanese PLWH in clinical practice. The results showed that patients in the study had a high rate of non-adherence when gaps of up to 4 weeks were allowed but a lower rate when gaps of up to 8 weeks were allowed. The study findings emphasized the importance of raising awareness among health care providers and thorough patient education to address non-adherence to denosumab. As this was a retrospective, exploratory study with a small sample size, further studies in larger PLWH populations are needed to identify factors affecting non-adherence to denosumab treatment.

### Supplementary Information

**Additional file 1:** **Supplemental Figure S1.** Subject Enrollment Flowchart. **Supplemental Figure S2.** Kaplan–Meier survival curve for denosumab treatment adherence and persistence. **Supplemental Figure S3.** Comparison of annualized lumbar spine and femoral neck BMD changes between denosumab treatment adherence group and non-adherence group at 24 months (*n*=28). **Supplemental Figure S4.** Correlations between annualized lumbar spine and femoral neck BMD changes and medication coverage ratio to follow-up (*n*=28).

**Additional file 2:** **Supplemental Table S1.** Demographic and clinical characteristics of initiated denosumab treatment before versus after the COVID-19 pandemic (*n*=29). ^a^

## Data Availability

All data generated or analyzed during this study are included in this published article and its supplementary information files.

## Abbreviations

HIV: Human immunodeficiency virus
ART: Antiretroviral therapy
PLWH: People living with HIV
ARV: Antiretroviral drug
TFV: Tenofovir
BMD: Bone mineral density
COVID-19: Coronavirus disease 2019
MCR: Medication coverage ratio
AE: Adverse event
INSTI: Integrase strand transfer inhibitor
