# Supplementary material for: Adherence of denosumab treatment for low bone mineral density in Japanese people living with HIV: a retrospective observational study
Source: J Pharm Health Care Sci. 2023 Dec 7;9:45. doi: 10.1186/s40780-023-00315-9 (PMC10702095; doi:10.1186/s40780-023-00315-9)
Supplement: Supplementary file 1 — Additional file 1: Supplemental Figure S1. Subject Enrollment Flowchart. Supplemental Figure S2. Kaplan–Meier survival curve for denosumab treatment adherence and persistence. Supplemental Figure S3. Comparison of annualized lumbar spine and femoral neck BMD changes between denosumab treatment adherence group and non-adherence group at 24 months (n=28). Supplemental Figure S4. Correlations between annualized lumbar spine and femoral neck BMD changes and medication coverage ratio to follow-up (n=28). [file 40780_2023_315_MOESM1_ESM.pptx]

## Slide 1
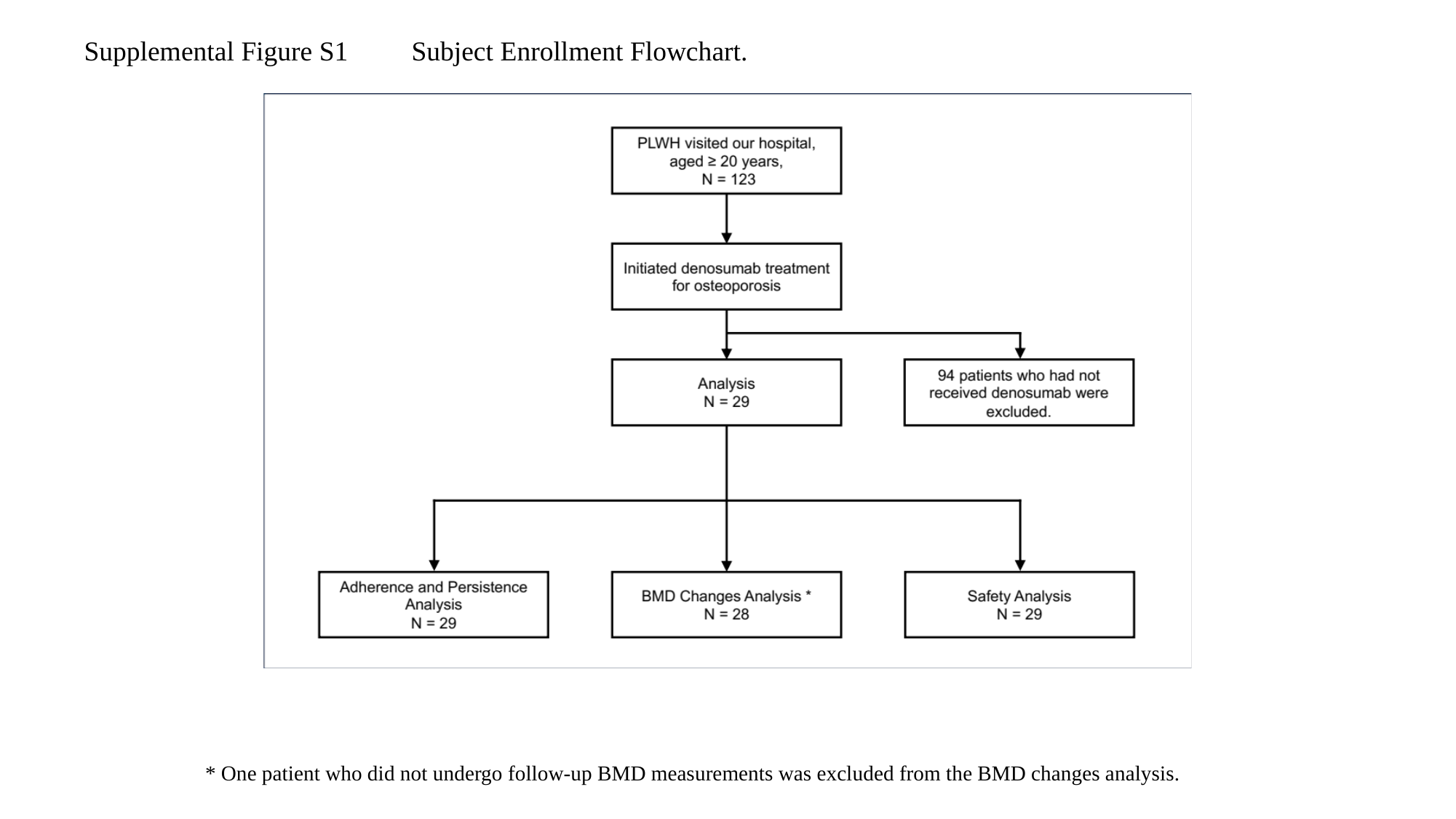

Supplemental Figure S1	Subject Enrollment Flowchart.
* One patient who did not undergo follow-up BMD measurements was excluded from the BMD changes analysis.

## Slide 2
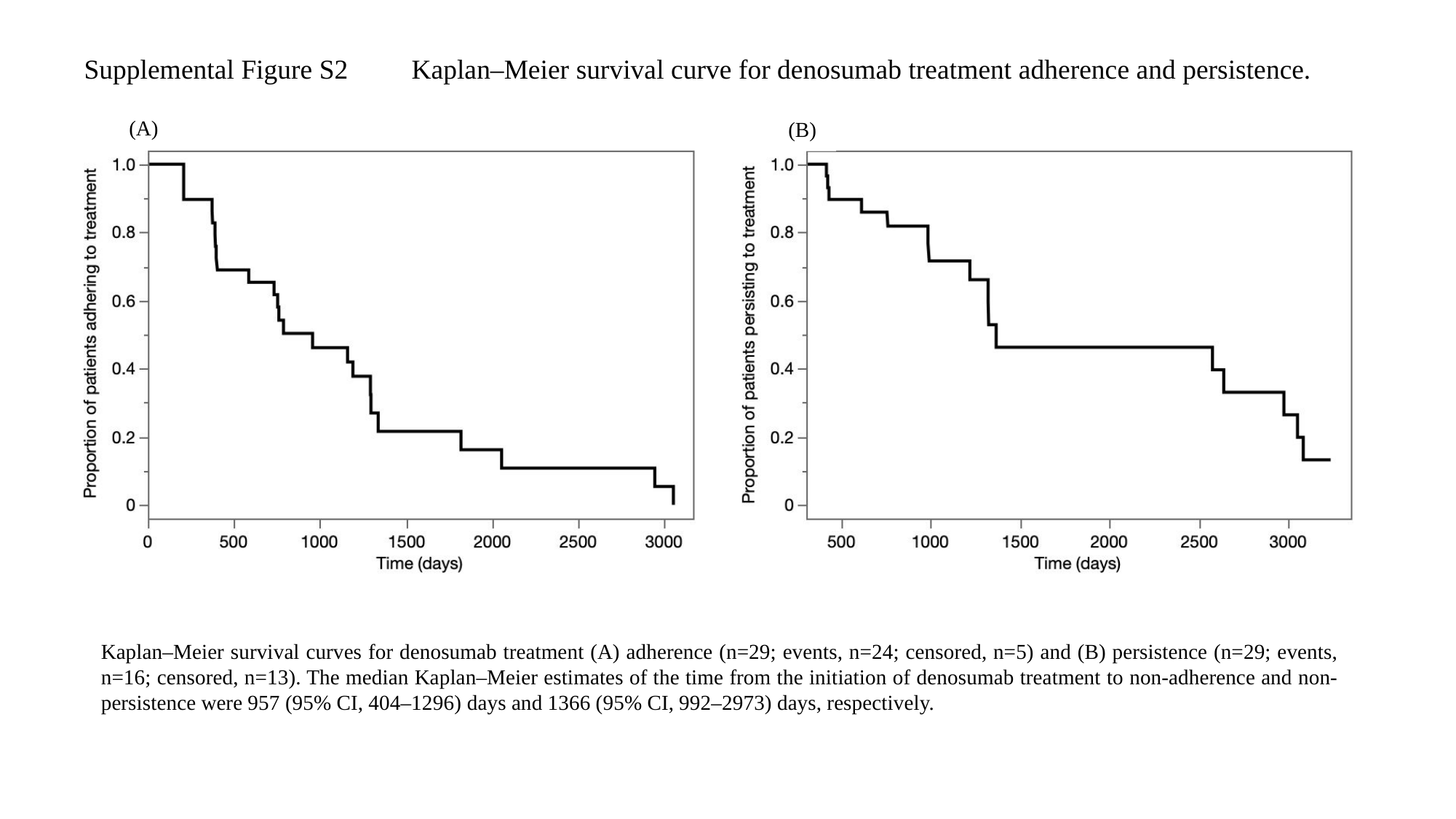

Supplemental Figure S2	Kaplan–Meier survival curve for denosumab treatment adherence and persistence.
(A)
(B)
Kaplan–Meier survival curves for denosumab treatment (A) adherence (n=29; events, n=24; censored, n=5) and (B) persistence (n=29; events, n=16; censored, n=13). The median Kaplan–Meier estimates of the time from the initiation of denosumab treatment to non-adherence and non-persistence were 957 (95% CI, 404–1296) days and 1366 (95% CI, 992–2973) days, respectively.

## Slide 3
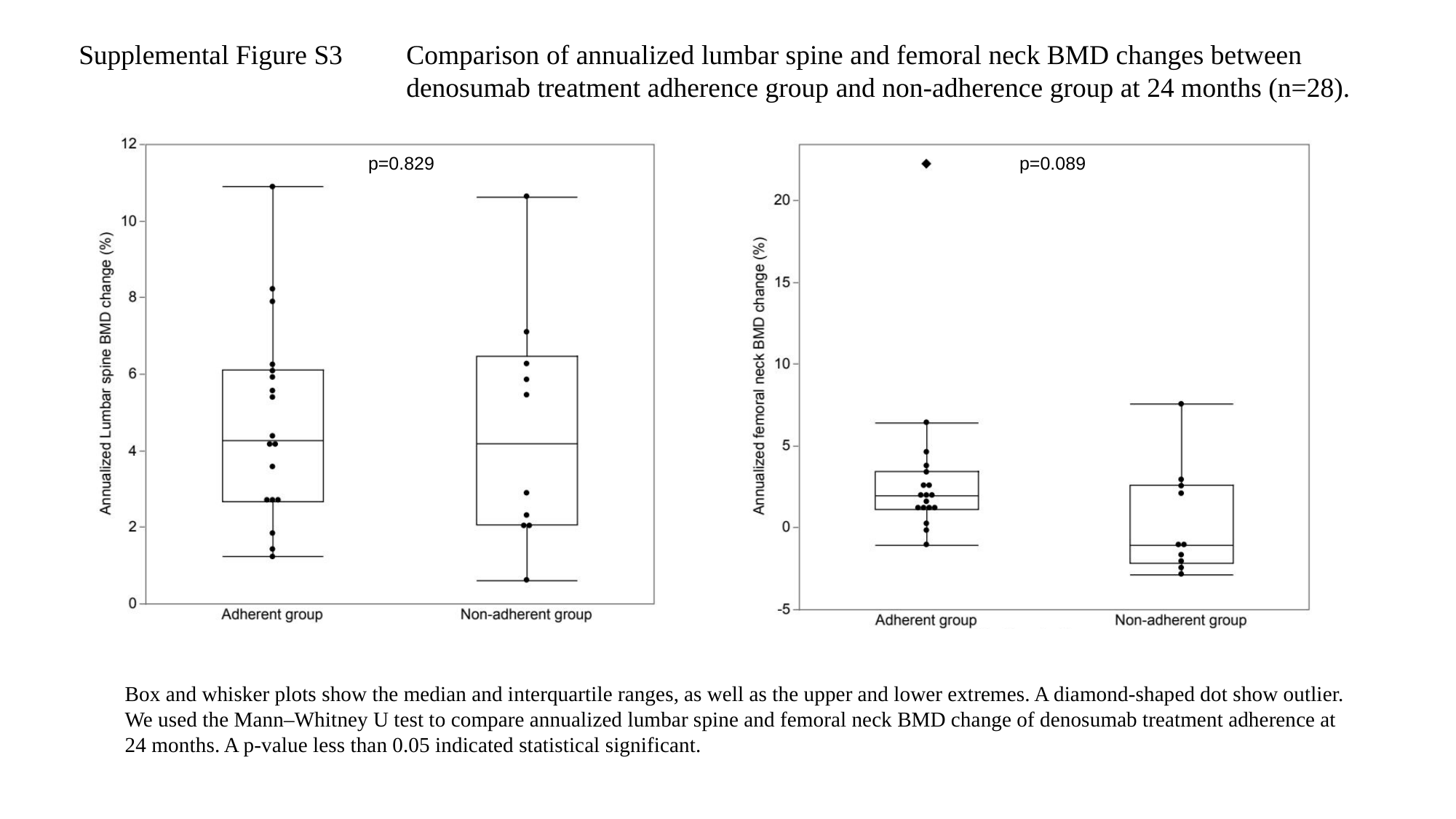

Supplemental Figure S3	Comparison of annualized lumbar spine and femoral neck BMD changes between 				denosumab treatment adherence group and non-adherence group at 24 months (n=28).
p=0.829
p=0.089
Box and whisker plots show the median and interquartile ranges, as well as the upper and lower extremes. A diamond-shaped dot show outlier. We used the Mann–Whitney U test to compare annualized lumbar spine and femoral neck BMD change of denosumab treatment adherence at 24 months. A p-value less than 0.05 indicated statistical significant.

## Slide 4
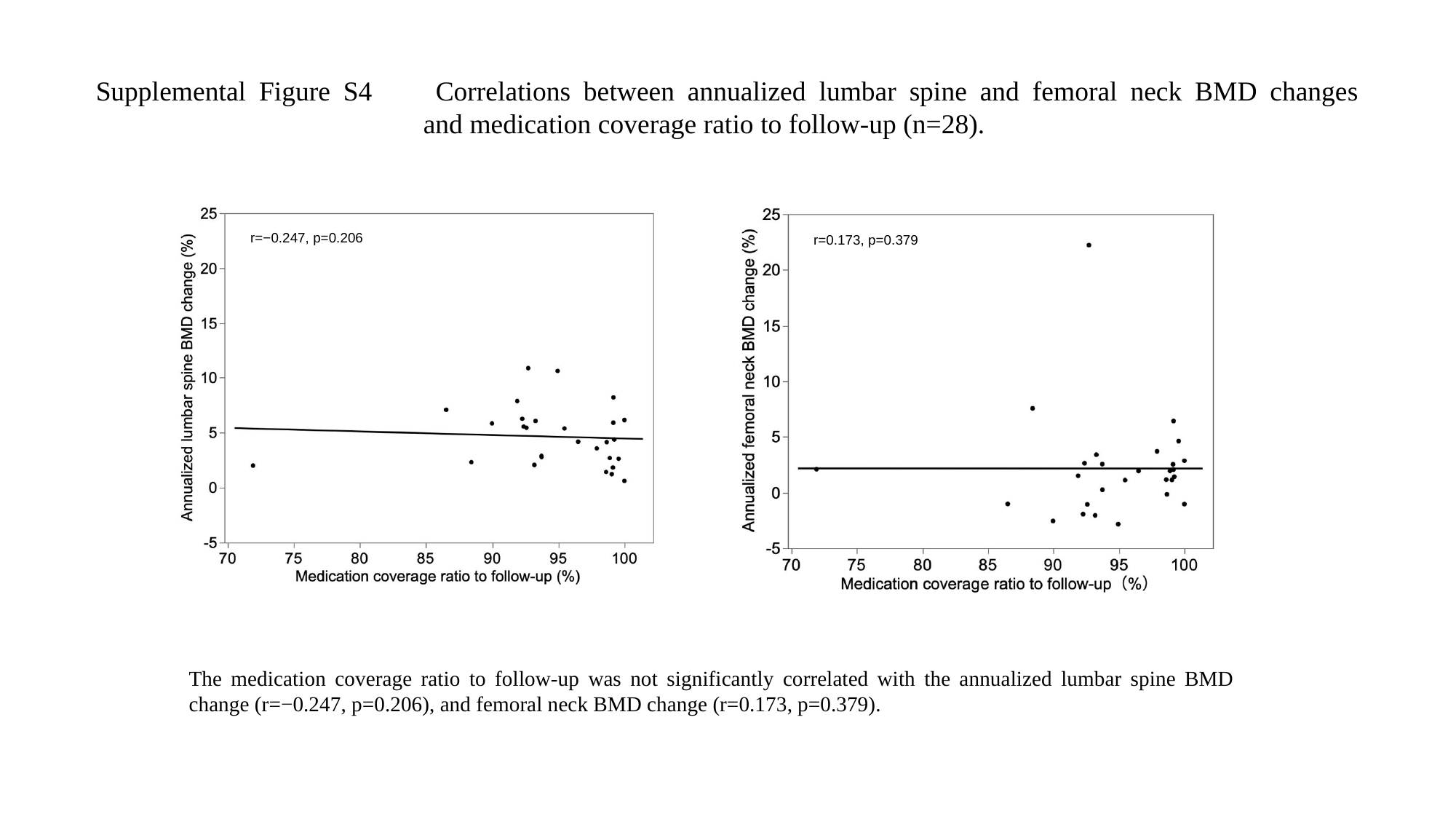

Supplemental Figure S4	Correlations between annualized lumbar spine and femoral neck BMD changes 			and medication coverage ratio to follow-up (n=28).
r=−0.247, p=0.206
r=0.173, p=0.379
The medication coverage ratio to follow-up was not significantly correlated with the annualized lumbar spine BMD change (r=−0.247, p=0.206), and femoral neck BMD change (r=0.173, p=0.379).
