# Supplementary material for: Adherence of denosumab treatment for low bone mineral density in Japanese people living with HIV: a retrospective observational study
Source: J Pharm Health Care Sci. 2023 Dec 7;9:45. doi: 10.1186/s40780-023-00315-9 (PMC10702095; doi:10.1186/s40780-023-00315-9)
Supplement: Supplementary file 2 — Additional file 2: Supplemental Table S1. Demographic and clinical characteristics of initiated denosumab treatment before versus after the COVID-19 pandemic (n=29). a [file 40780_2023_315_MOESM2_ESM.docx]

Supplemental Table S1 Demographic and clinical characteristics of initiated denosumab treatment before versus after the COVID-19 pandemic (n=29). ^a^

| Characteristics | Initiated denosumab treatment | | p-value ^b^ |
| --- | --- | --- | --- |
|  | Before the COVID-19 pandemic (n=22) | After the COVID-19 pandemic (n=7) |  |
| Age (years), median (IQR) | 39.0 (35.8­–44.0) | 38.0 (34.0­–45.0) | 0.721 |
| Gender, male, n (%) | 20 (90.9) | 6 (85.7) | 1.000 |
| BMI (kg/m^2^), median (IQR) | 20.3 (18.6­–23.7) | 19.6 (18.8–23.7) | 0.980 |
| Current smoking, n (%) | 8 (36.4) | 4 (57.1) | 0.403 |
| Prior AIDS diagnosis, n (%) | 6 (27.3) | 4 (57.1) | 0.193 |
| Time since diagnosis HIV (years), median (IQR) | 5 (1–9) | 1 (0–1) | 0.052 |
| CD4 cell count (cells/µL), median (IQR) | 545 (374–828) | 154 (133–425) | 0.012 |
| Time on antiretroviral therapy (years), median (IQR) | 4 (2–9) | 1 (1–1) | 0.022 |

Abbreviations: AIDS, acquired immunodeficiency syndrome; BMI, body mass index; COVID-19, coronavirus disease 2019; HIV, human immunodeficiency virus; IQR, interquartile range;

^a^ Data are expressed as number and frequency (%) or median value with interquartile range (IQR).

^b^ Fisher’s exact test was used for categorical data, and the Mann–Whitney U test was used for continuous data.
